# Trastuzumab Deruxtecan Improves Progression-Free Survival and Intracranial Response in Patients with HER2-Positive Metastatic Breast Cancer and Brain Metastases

**DOI:** 10.1093/oncolo/oyac009

**Published:** 2022-03-28

**Authors:** Anne Jacobson

## Abstract

In the phase III DESTINY-Breast03 trial, trastuzumab deruxtecan improved progression-free survival (PFS) relative to trastuzumab emtansine across all patients with HER2-positive metastatic breast cancer, including a 75% improvement in PFS among those with baseline brain metastases.

Human epidermal growth factor receptor 2 (HER2)-positive breast cancer often progresses after standard first-line therapy, creating a need for effective second-line treatment options. In 2019, the U.S. Food and Drug Administration approved fam-trastuzumab deruxtecan-nxki (T-DXd) for the treatment of patients with unresectable or metastatic HER2-positive breast cancer who have received two or more prior HER2-targeted regimens.

The phase III DESTINY-Breast03 trial is the first head-to-head trial comparing T-DXd with trastuzumab emtansine (T-DM1) in patients with HER2-positive metastatic breast cancer in the second-line setting, after prior treatment with trastuzumab and a taxane.^1,2^

As reported at the 2021 European Society for Medical Oncology (ESMO) annual meeting, initial results from DESTINY-Breast03 showed a 72% improvement in progression-free survival (PFS) with T-DXd compared with T-DM1.^1^ The 12-month PFS rate was 75.8% and 34.1% in the T-DXd and T-DM1 groups, respectively (HR, 0.28; *p* <.0001).

In the current analysis, Sara A. Hurvitz, M.D., of the David Geffen School of Medicine at the University of California, Los Angeles, presented findings from key patient subgroups in the DESTINY-Breast03 trial, including patients with brain metastases.^2^

## Study Design

The international DESTINY-Breast03 trial enrolled 524 patients with HER2-positive unresectable or metastatic breast cancer who were previously treated with trastuzumab and a taxane in the advanced or metastatic setting. Patients were randomly assigned to treatment with T-DXd (*n* = 261) or T-DM1 (*n* = 263). The primary endpoint was PFS.

Baseline characteristics were similar in both treatment groups. The median patient age was 54 years. Approximately 50% of patients were treated with 0-1 prior lines of therapy in the metastatic setting, and 50% were treated with ≥2 prior treatment regimens. At baseline, 16.5% of patients in the T-DXd group and 14.8% of those in the T-DM1 group had brain metastases.

## Key Findings

At a median follow-up of 15.9 months, T-DXd significantly improved PFS by 72% compared with T-DM1 across all patient subgroups (Table 1). Findings were consistent irrespective of hormone receptor status, prior treatment with pertuzumab, number of prior lines of therapy, presence or absence of visceral disease, and presence or absence of brain metastases.

**Table 1.** Progression-free survival in HER2-positive metastatic breast cancer

**Patient subgroup**	**T-DXd**	**T-DM1**	**HR (95% CI)**
All patients (*N* = 524)	Not reached	6.8 months	0.28 (0.22-0.37)
Hormone receptor status			
Positive (*n* = 272)	22.4 months	6.9 months	0.32 (0.22-0.46)
Negative (*n* = 248)	Not reached	6.8 months	0.30 (0.20-0.44)
Prior pertuzumab treatment			
Yes (*n* = 320)	Not reached	6.8 months	0.32 (0.22-0.46)
No (*n* = 204)	Not reached	7.0 months	0.30 (0.19-0.47)
Prior lines of therapy			
0-1 (*n* = 258)	22.4 months	8.0 months	0.33 (0.23-0.48)
≥2 (*n* = 266)	Not reached	5.6 months	0.28 (0.19-0.41)
Visceral metastases			
Yes (*n* = 384)	22.2 months	5.7 months	0.28 (0.21-0.38)
No (*n* = 140)	Not reached	11.3 months	0.32 (0.17-0.58)
Brain metastases			
Yes (*n* = 82)	15.0 months	3.0 months	0.25 (0.13-0.45)
No (*n* = 442)	Not reached	7.1 months	0.32 (0.22-0.40)

Abbreviations: CI, confidence interval; HER2, human epidermal growth factor receptor 2; HR, hazard ratio; T-DM1, trastuzumab emtansine; T-DXd, trastuzumab deruxtecan.

The overall response rate (ORR) in the overall study cohort was 79.7% and 34.2% in the T-DXd and T-DM1 groups, respectively, representing an absolute improvement in ORR of 45% with T-DXd. Findings were consistent across all patient subgroups, with the absolute improvement in ORR associated with T-DXd relative to T-DM1 ranging from 39% to 52%.

## Disease Control in Patients with Brain Metastases

Treatment with T-DXd significantly improved PFS, ORR, and intracranial response in patients with brain metastases at baseline (*n* = 82). In this subgroup, T-DXd reduced the risk of disease progression or death by 75% relative to T-DM1. The 12-month PFS rate in this subgroup was 72.0% with T-DXd and 20.9% with T-DM1 (HR, 0.27; 95% CI, 0.13-0.45).

Among those with brain metastases, the ORR was 67.4% and 20.5% in the T-DXd and T-DM1 treatment groups, respectively. The median duration of response was 12.9 months with T-DXd and 7.2 months with T-DM1.

In the assessment of intracranial response, 27.8% of patients treated with T-DXd achieved a complete intracranial response, compared with 2.8% of patients treated with T-DM1. The overall intracranial response for patients with brain metastases was 63.8% in the T-DXd group and 33.3% in the T-DM1 group.

## Safety

Treatment with T-DXd was associated with a manageable safety profile, with a similar rate of all adverse events (AEs) and grade ≥3 AEs compared with T-DM1. The most common grade ≥3 AEs in the T-DXd group were neutropenia (19.1%), thrombocytopenia (7.0%), nausea (6.6%), leukopenia (6.6%%), anemia (5.8%), and fatigue (5.1%). There were no grade 4 or 5 cases of interstitial lung disease or pneumonitis in either treatment group.

In summary, T-DXd demonstrated a statistically significant and clinically meaningful improvement in PFS and ORR compared with T-DM1 across all patient groups, including those with baseline brain metastases. These findings support the use of T-DXd as a standard of care for patients with previously treated HER2-positive metastatic breast cancer.

## Notes

Please note that this content was created for the purpose of summarizing the content of the San Antonio Breast Cancer Symposium 2021 and has not been peer reviewed under the standard policies of *The Oncologist*.
